# TDO2 Was Downregulated in Hepatocellular Carcinoma and Inhibited Cell Proliferation by Upregulating the Expression of p21 and p27

**DOI:** 10.1155/2021/4708439

**Published:** 2021-08-13

**Authors:** Chengpeng Yu, Dean Rao, He Zhu, Qiumeng Liu, Wenjie Huang, Long Zhang, Huifang Liang, Jia Song, Zeyang Ding

**Affiliations:** ^1^Hepatic Surgery Center, Tongji Hospital, Tongji Medical College, Huazhong University of Science and Technology, Wuhan, China; ^2^Hubei Key Laboratory of Hepato-Pancreato-Biliary Diseases, Tongji Hospital, Tongji Medical College, Huazhong University of Science and Technology, Wuhan, China; ^3^Department of Hepato-Pancreato-Biliary Surgery, Ganzhou People's Hospital of Jiangxi Province (Ganzhou Hospital Affiliated of Nanchang University), Ganzhou Jiangxi 431000, China

## Abstract

**Background:**

Tryptophan-2,3-dioxygenase (TDO2) converts tryptophan into kynurenine in the initial limiting step of the kynurenine pathway. During the past decade, the overexpression of TDO2 has been found in various human tumors. However, the role of TDO2 in hepatocellular carcinoma is controversial, and we sought to clarify it in this study.

**Methods:**

Western blot analysis and immunochemistry were used to detect the expression of TDO2 in human tissue specimens. The effect of TDO2 on cell proliferation in vitro was assessed using CCK8 and colony formation assays, and a xenograft mouse model was used to detect the effect of TDO2 on tumor growth in vivo. Flow cytometry was used to assess the cell cycle status.

**Results:**

Low TDO2 expression was found in HCC and was associated with poor prognosis and adverse clinical outcomes. Conversely, TDO2 could restrain the proliferation of HCC cells in vivo and in vitro. Furthermore, TDO2 upregulated the expression of p21 and p27, inducing cell-cycle arrest.

**Conclusions:**

The loss of TDO2 expression in HCC was correlated with a poor prognosis and adverse clinical outcomes. At the same time, TDO2 could restrain the growth of HCC in vivo and in vitro. The results indicate that TDO2 is a potential biomarker and therapeutic target for HCC.

## 1. Introduction

As the sixth most commonly diagnosed type of cancer worldwide, hepatocellular carcinoma (HCC) imposes a heavy burden on global health [1]. HCC is an aggressive cancer and often diagnosed at an advanced stage, which results in a poor prognosis because surgical resection is only effective in the early stage [2]. Although combining immune checkpoint inhibitors with antivascular endothelial growth factor (VEGF) antibodies demonstrated a promising survival benefit for some patients with advanced HCC, this combined treatment is not effective for all advanced HCC patients [3, 4]. Hence, it is vital to explore and understand the complex pathogenesis of HCC to develop new therapeutic targets and biomarkers for the early diagnosis and treatment of HCC.

Tryptophan-2,3-dioxygenase (TDO2) converts tryptophan into kynurenine in the initial limiting step of the kynurenine pathway [5]. Under physiological conditions, TDO2 is only expressed in the liver, where it is responsible for tryptophan metabolism. However, TDO2 can also be expressed in other tissues, such as the placenta, epididymis, and testes [6, 7]. During the past decade, TDO2 was found to be overexpressed in various tumors, such as glioma, breast cancer, basal cell carcinoma, melanoma, cervical cancer, and colorectal cancer [5, 8–13]. As the depletion of tryptophan and the production of its downstream metabolites can restrain the proliferation of immune cells, the overexpression of TDO2 in tumors was considered to promote tumor immune escape and stimulate cancer metastasis [5, 12, 14]. However, the role of TDO2 in hepatocellular carcinoma is controversial. Some studies reported that TDO2 is highly expressed in hepatocellular carcinoma [15, 16], while others implied that TDO2 in HCC tumors is expressed at lower levels than in adjacent tissues [5, 17].

In this study, we firstly used UALCAN (http://ualcan.path.uab.edu) to conveniently access clinical data from TCGA for an analysis of the differences in *TDO2* mRNA levels between normal samples and HCC as well as their relationship to clinicopathological parameters [18]. The results revealed lower *TDO2* mRNA levels in HCC than in normal tissues. We then examined TDO2 protein expression in a clinical tissue microarray (TMA) and tissue specimens of HCC using immunohistochemical (IHC) staining and western blot analysis, which revealed lower TDO2 protein levels in tumor specimens than in normal tissues. At the same time, Kaplan-Meier survival analysis showed that the loss of TDO2 expression was associated with shorter survival of HCC patients. Furthermore, we also analyzed the roles of TDO2 in HCC cell lines both in vivo and in vitro. Overexpression of TDO2 restrained the proliferation of HCC cells, resulting in cell-cycle arrest in the G1 phase. Additionally, TDO2 was found to upregulate the expression of p21 and p27 while reducing CDK2 and CDK4 expression.

## 2. Patients and Methods

### 2.1. Patients

Primary HCC tissues and adjacent matched normal tissues from a total of 116 patients who underwent tumor resection at the Hepatic Surgery Center, Tongji Hospital of Huazhong University of Science and Technology (HUST) (Wuhan, China) between 2012/2/16 and 2014/4/1 were used in this study. In addition, 40 paired fresh specimens of HCC tissues and adjacent normal tissues from the specimen collection were used for western blot analysis. Overall survival (OS) and disease-free survival (DFS) was defined as the time from the first surgery to death or recurrence/metastasis, respectively.

### 2.2. Western Blot Analysis

Cold RIPA lysis buffer (Boster Biological Technology, Wuhan, Hubei, China) with a protease inhibitor cocktail was used to extract total protein from tissue samples. The protein concentration was measured by using a bicinchoninic acid (BCA) protein quantification kit (Wuhan Boster Biological Technology, Ltd., Wuhan, China). The proteins were separated on a 10% acrylamide SDS-PAGE gel and transferred to a PVDF membrane (Merck Millipore, Billerica, MA). Then, the membrane was blocked with 5% BSA and incubated with appropriately diluted primary antibodies against TDO2 (#H00006999-D01P, Abnova), p21 (#2947, Cell Signaling Technology), p27 (#3686, Cell Signaling Technology), p53 (#2524, Cell Signaling Technology), CDK2 (sc-748, Santa Cruz), CDK4 (#1290, Cell Signaling Technology), and GAPDH (KC-5G4; KangChen BioTech Co., Ltd.) overnight at 4°C. The membrane was then washed with phosphate-buffered saline (PBS) and incubated with HRP-labelled goat anti-rabbit or anti-mouse secondary antibodies (ab6702, ab205719, 1 : 2000, Abcam) for 1 h at room temperature. An enhanced chemiluminescence (ECL) reagent (Merck Millipore, Billerica, MA) was used to develop the blots. Image Lab Software was used to quantify the grayscale density of the protein bands.

### 2.3. Immunohistochemistry

Paraffin sections of 4 *μ*m thickness were used for TDO2 detection. Xylene and a gradient of ethanol solutions were used to dewax and gradually hydrate the tissue sections. For antigen retrieval, the slides were heated in a steam cooker for 10–15 min in 0.01 mol/l citrate buffer (pH 6.0). Then, the slides were washed with PBS (pH 7.4), and endogenous peroxidases were blocked by incubation in 1 × TBS with 0.3% H_2_O_2_ for 20 min at room temperature. Then, the primary anti-TDO2 polyclonal antibody was added to the samples and incubated at 4°C overnight. On the next day, 1 × TBS was used to wash the slides thoroughly, and a fresh substrate solution containing diaminobenzidine was used to visualize the antibody binding. Two independent pathologists who were blinded to the patient information assessed the immunohistochemical staining. Each specimen was assessed and scored according to the percentage of stained cells (0–5% = 0, 5–25% = 1, 26–50% = 2, 51–75% = 3, and 76–100% = 4) and intensity of positive tumor cells (none = 0, weak = 1, intermediate = 2, and strong = 3) and summed up to calculate the immunostaining score of TDO2 expression. The low- and high-level expressions were judged based on overall scores of <6 and ≥6, respectively.

### 2.4. Flow Cytometry

Propidium Iodide Staining Solution (BD Pharmingen™, New Jersey, USA) was used for flow cytometry. Cells were trypsinized and suspended at 1 × 10^5^ cells/ml, followed by staining with a fluorescent antibody and propidium iodide (PI) at room temperature in the dark for 30 min. After incubation, the cells were centrifuged at 200 g and resuspended for analysis on a FACS instrument (BD Bioscience, San Jose, CA).

### 2.5. Cell Lines and Culture Conditions

HLF cells were obtained from our laboratory, while HEK293 and HepG2 cells were ordered from the China Center for Type Culture Collection (CCTCC, Wuhan, China). All cells were cultured at 37°C in Dulbecco's Modified Eagle's Medium (DMEM) (high glucose) (HyClone, USA) supplemented with 10% fetal bovine serum (Bovogen, Argentina) in a humidified atmosphere comprising 5% CO_2_.

### 2.6. Plasmids and Lentivirus

The plenti-CMV-Puro, pMD2.G, gagpol, and psPAX2 plasmids (Addgene plasmids #17448 # 12259, #35614 and 12260; Addgene, Cambridge, MA, USA) were used for gene expression and lentivirus packaging. The CDS of the *tdo2* gene was amplified by PCR and subcloned into the lentiviral vector plenti-CMV-Puro. HEK293 cells were cotransfected with psPAX2 and pMD2.G, pLenti-CMV-Puro, or pLenti-CMV-TDO2-Puro to produce lentivirus virions. We collected the virions in the supernatant at 48 and 72 hr posttransfection and passed them through a 0.45 *μ*m filter before storage at -80°C. The resulting lentivirus stock was used to transfect HLF and HepG2 cells to establish stable cell lines.

### 2.7. Cell Proliferation and Colony Formation Assays

A total of 1000 cells in 100 *μ*l of medium was seeded into each well of the 96-well plate. After culturing for 24, 48, 72, 96, and 120 h, 100 *μ*l of CCK8 solution (Cell Counting Kit-8; Beyotime Institute of Biotechnology, Shanghai, China) in each well was incubated for 2 h, followed by measurement of the absorbance at 450 nm using a microplate reader (BioTek, Winooski, VT, USA). For the colony formation assay, each well of a 6-well plate was seeded with 1000 cells, which were cultured for 2 weeks at 37°C. The resulting colonies were fixed with 20% methanol and stained using 0.1% crystal violet, followed by manual counting under a conventional optical microscope. All experiments were done in triplicate.

### 2.8. Tumor Growth Assay In Vivo

All animal experiments were conducted with the approval of the Ethic Committee of Tongji Hospital of HUST and were in accordance with the animal welfare guidelines of the People's Republic of China National Standard (GB/T 35,892-2018). For the xenograft model, 4-week-old male BALB/c athymic nude mice were randomly divided into 2 groups (*n* = 6 per group) and injected subcutaneously with 1.5 × 106 tumor cells in 100 *μ*l of serum-free DMEM into the flank. Every 3 days, the development of the tumors was observed, and the mice were sacrificed after the indicated number of days. The tumors were then removed, photographed, weighed, and measured.

### 2.9. Statistical Analysis

Statistical analyses were conducted using SPSS 22.0 (IBM Corp., USA). The *χ*^2^ test was used to evaluate the statistical significance of the correlation between clinicopathological variables and TDO2 expression.

## 3. Results

### 3.1. The Loss of TDO2 Expression in HCC Is Related to Poor Clinical Outcomes

**Figure 1:**
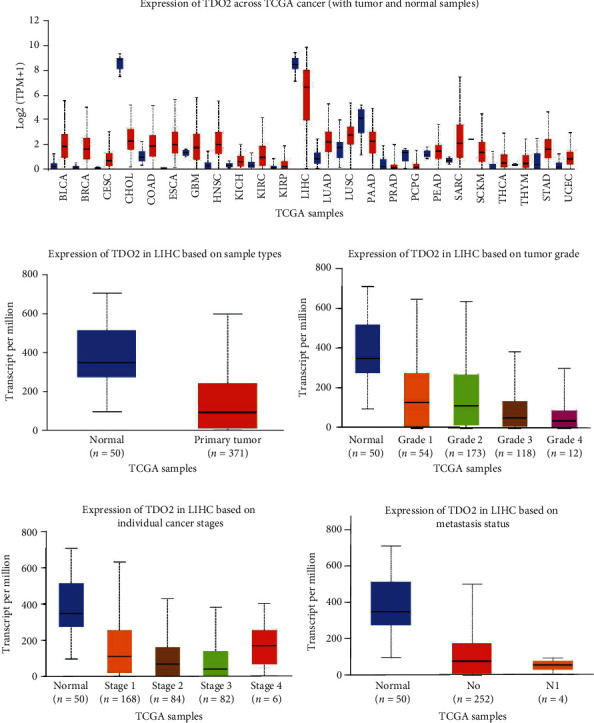
The mRNA expression of TDO2 in HCC samples from TCGA, according to UALCAN analysis. (a) The mRNA expression of TDO2 across different cancers in TCGA. (b) The mRNA expression of TDO2 in liver cancer stratified by sample type. (c) The expression of TDO2 in liver cancer stratified by tumor grade. (d) The expression of TDO2 in liver cancer stratified by cancer stage. (e) The expression of TDO2 in liver cancer stratified by nodal metastasis status.

**Figure 2:**
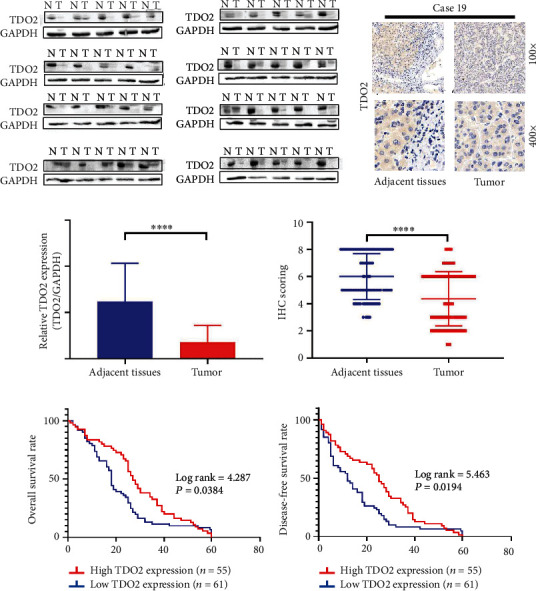
TDO2 was downregulated in HCC. (a) Western blot analysis of TDO2 expression in 40 paired fresh specimens of HCC and normal tissues. (b) Immunochemistry analysis of TDO2 expression in 116 pairs of primary HCC tissues and adjacent normal tissues. (c) Column chart of western blot analysis of TDO2 expression in 40 paired fresh specimens of HCC tissues and adjacent normal tissues. (d) Dot chart of immunochemistry analysis of TDO2 expression in 116 pairs of primary HCC tissues and adjacent normal tissues. (e) Kaplan-Meier analysis of overall survival in patients with high and low TDO2 expression. (f) Kaplan-Meier analysis of disease-free survival in patients with high and low TDO2 expression.

In spite of its widely acknowledged effects in many other cancers, the role of TDO2 in HCC is controversial. Consequently, we first used data from TCGA to detect the expression of TDO2 in HCC. UALCAN was used to identify TDO2 mRNA levels in 24 types of cancers compared to normal tissues in TCGA [18]. As shown in Figures 1(a) and 1(b), the expression of TDO2 was upregulated in most cancers, but it was downregulated in cholangiocarcinoma, hepatocellular carcinoma, and pancreatic adenocarcinoma (Figures 1(a) and 1(b)). As TDO2 is only expressed in the liver under physiological conditions, we wished to explore the expression of TDO2 in HCC. We used 40 paired fresh HCC specimens and adjacent normal tissues for western blot and tissue microarray analyses of the expression of TDO2. The results indicated that TDO2 expression was downregulated in HCC (Figures 2(a)–2(d)). Furthermore, we used UALCAN to analyze the relationship between TDO2 expression and clinicopathological characteristics in HCC, which revealed that low TDO2 expression was correlated with a low tumor grade, advanced cancer stage, and cancer metastasis (Figures 1(c)–1(e)). Then, TDO2 expression in tumors from 116 HCC patients was assessed by microarray IHC analysis. Subsequently, the patients were divided into low- and high-expression groups according to the immunostaining score to explore the relationship between TDO2 expression and clinical outcomes. The results of regression analysis indicated that low TDO2 expression was associated with tumor size (*P* = 0.011), tumor stage (BCLC stage *P* = 0.019 and TNM stage *P* = 0.027), tumor differentiation (*P* = 0.038), and tumor recurrence (*P* = 0.041). However, low TDO2 expression was not associated with age, sex, ALT level, AST level, serum AFP level, tumor number, and tumor capsule (Table 1). Additionally, Kaplan-Meier survival analysis was used to investigate the relationship between the expression of TDO2 and overall survival (OS) or disease-free survival (DFS). The results demonstrated that patients with low TDO2 expression had shorter OS (*P* = 0.0384) (Figure 2(e)) and DFS (*P* = 0.0194) (Figure 2(f)).

### 3.2. Overexpression of TDO2 Inhibited Cell Proliferation In Vitro

**Figure 3:**
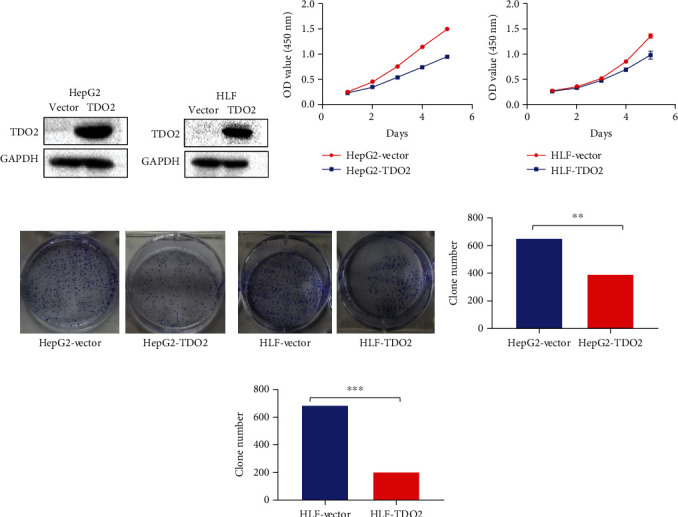
Overexpression of TDO2 restrained the proliferation of HepG2 and HLF cells. (a, b) Western blot showing that TDO2 was overexpressed in transfected HLF and HepG2 cells. (c, d) CCK8 assay showing that overexpression of TDO2 could restrain the proliferation of HLF and HepG2 cells. (e, f) Overexpression of TDO2 reduced the colony formation of HLF and HepG2 cells. (g, h) Column chart of the colony formation of HLF and HepG2 cells stably transfected with a TDO2-overexpression vector or the empty vector control.

Since the loss of TDO2 expression was correlated with adverse clinical outcomes and a poor prognosis in HCC, it is possible that TDO2 acts as a tumor suppressor. Accordingly, we wanted to investigate the effect of TDO2 on tumorigenesis in HCC cell lines and used lentiviral vectors to transduce HLF and HepG2 cells to establish stable cell lines with TDO2 overexpression. The expression of TDO2 was detected by WB (Figures 3(a) and 3(b)). The CCK8 assay indicated that TDO2 overexpression restrained the proliferation of HLF and HepG2 cells (Figures 3(c) and 3(d)). At the same time, the overexpression of TDO2 in HLF and HepG2 cells reduced their colony formation ability (Figures 3(e) and 3(f)). Taken together, these results show that TDO2 restrained the proliferation of HCC cell lines in vitro.

### 3.3. Overexpression of TDO2 Induced Cell Cycle Arrest and Upregulated the Expression of p21 and p27 In Vitro

**Figure 4:**
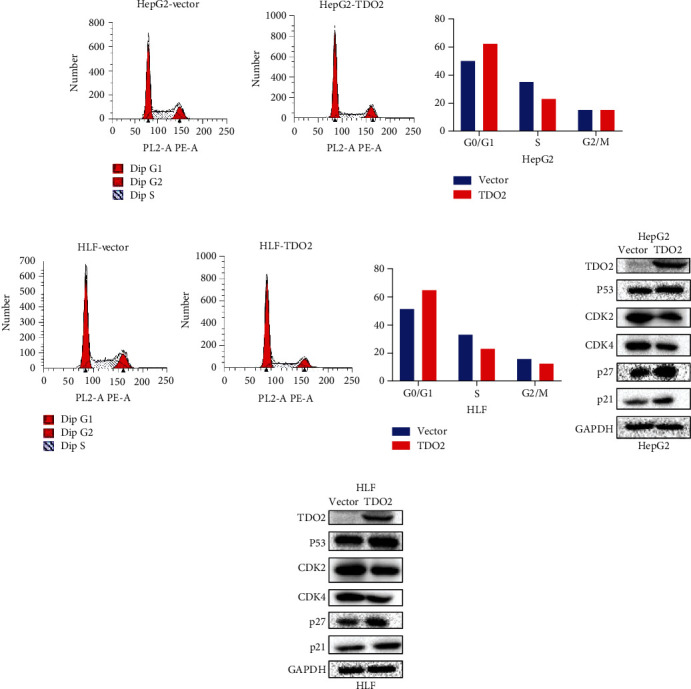
(a, b) Flow cytometry analysis showing that overexpression of TDO2 increased the percentage of HepG2 cells in the G1 phase of the cell cycle. (c, d) Flow cytometry analysis showing that overexpression of TDO2 increased the percentage of HLF cells in the G1 phase. (e, f) Overexpression of TDO2 increased the expression of p21 and p27 while decreasing the expression of CDK2 and CDK4 in HepG2 and HLF cells.

As the results of the CCK8 and colony formation assays demonstrated that the overexpression of TDO2 could restrain the proliferation of HCC cell lines, flow cytometry and western blot analyses were used to explore the effects of TDO2 on the cell cycle and related proteins, respectively. The results of flow cytometry demonstrated that the overexpression of TDO2 increased the percentage of cells in the G1 phase in both the HLF and HepG2 HCC cell lines, leading to cell cycle arrest (Figures 4(a)–4(d)). Furthermore, the results of western blot analysis showed that the overexpression of TDO2 enhanced the expression of p21 and p27 while reducing the expression of CDK2 and CDK4 (Figures 4(e) and 4(f)). In conclusion, the results indicate that TDO2 might induce cell cycle arrest via the upregulation of p21 and p27 expression.

### 3.4. TDO2 Overexpression Inhibited Tumor Growth In Vivo

**Figure 5:**
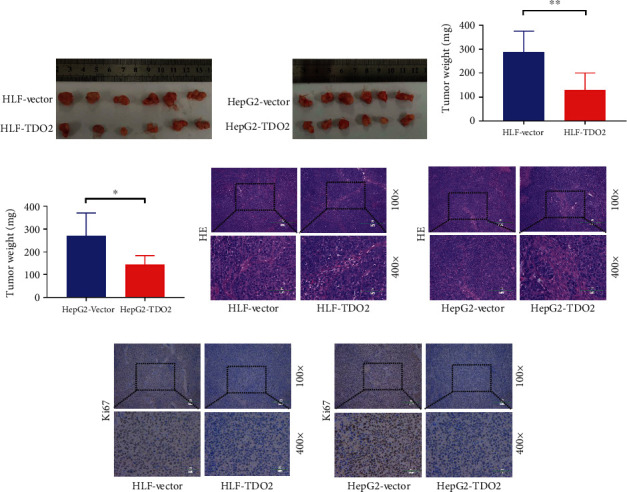
(a, b) Macroscopic images of the xenograft tumors comprising HLF and HepG2 line cells stably transfected with a TDO2-overexpression vector or the empty vector control. (c, d) Column chart of the weight of the xenograft tumors comprising HLF or HepG2 cells stably transfected with a TDO2-overexpression vector or the empty vector control. (e, f) Representative photographs of HE staining of the xenograft tumors comprising HLF or HepG2 cells stably transfected with a TDO2-overexpression vector or the empty vector control. (g, h) IHC staining for Ki67 expression in the xenograft tumors comprising HLF or HepG2 line cells stably transfected with a TDO2-overexpression vector or the empty vector control.

In order to further verify the influence of TDO2 on tumor growth in vivo, we subcutaneously injected HLF and HepG2 cells stably transfected with the TDO2 expression vector or an empty vector into nude mice. At 21 days after subcutaneous injection, the growth and weight of tumors comprising the TDO2-overexpressing stable cell lines were reduced compared to those of the empty vector group (Figures 5(a)–5(d)). At the end of the experiment, the subcutaneous tumor tissues were subjected to HE staining for histopathological assessment. Representative photographs of HE staining are shown in Figures 5(e) and 5(f). Furthermore, IHC was used to detect the expression of the cell proliferation marker Ki67 in the subcutaneous tumor tissue. The IHC staining showed that the expression of Ki67 in the TDO2 overexpression group was lower than that in the control group (Figures 5(g) and 5(h)).

## 4. Discussion

TDO2 is a heme-containing enzyme responsible for the oxidation of tryptophan [5]. Under physiological conditions, TDO2 is only expressed in the liver, where it is responsible for tryptophan metabolism [19]. Since TDO2 was firstly reported to be overexpressed in glioma, where it plays a tumor promoting role, increasing numbers of studies reported that TDO2 was overexpressed in other tumors, such as breast cancer, basal cell carcinoma, melanoma, cervical cancer, and colorectal cancer, where it is also involved in tumor development [5, 10, 13, 14]. However, the role of TDO2 in HCC is controversial. Some studies implied that TDO2 was downregulated in HCC [5, 17], while other studies indicated that TDO2 may be overexpressed in HCC [15]. In this study, we firstly used UALCAN to analyze the relative TDO2 mRNA levels in normal samples and HCC tumors and found that TDO2 mRNA expression in HCC samples was lower than in healthy tissues. Furthermore, we used WB and IHC to detect the expression of TDO2 in 40 paired fresh specimens of HCC and a tissue microarray, respectively, which confirmed that TDO2 was downregulated in HCC. At the same time, the loss of the TDO2 expression was correlated with a poor prognosis and adverse clinical outcomes. These results implied that TDO2 may function as a tumor suppressor in HCC, which is in contrast to the current viewpoint that TDO2 functions as a tumor promoter. However, it should be noted that TDO2 is normally only expressed in the liver where it is responsible for tryptophan metabolism. This in turn may indicate that its role in liver cancer is different from other tumors. Alternatively, the inconsistency of antibody staining and the cutoffs used to assign patients to high or low TDO2 expression groups might account for this discrepant finding.

As the loss of TDO2 was correlated with a poor prognosis and adverse clinical outcomes in HCC, we further explored the effect of TDO2 on HCC cell proliferation. We found that TDO2 could restrain the proliferation of HCC line cells and induce cell-cycle arrest by enhancing the expression of p21 and p27 while decreasing CDK2 and CDK4 expression. As most studies on the roles of TDO2 in tumors focus on tumor immune evasion, there are relatively few studies on the effect of TDO2 on the proliferation of tumor cells. As the depletion of tryptophan and accumulation of kynurenine can restrain the proliferation of T cells, TDO2 expression was also found to restrain the proliferation of T cells [5]. At the same time, IDO1, the isozyme of TDO2, was reported to induce cell cycle arrest by upregulating the expression of p27 via the kynurenine-AHR axis [20]. Accordingly, TDO2 might upregulate the expression of p21 and p27 via the kynurenine-AHR axis, but this conjecture needs further investigation.

Under normal physiological conditions, TDO2 is only expressed in the liver, while the expression of TDO2 is lost in HCC. At the same time, the expression of TDO2 is upregulated in most cancer types [9, 10, 13, 14]. Hence, the underlying mechanisms and effects to the loss of TDO2 expression in HCC merit further attention. As the promoter of the *tdo2* gene contains two glucocorticoid-responsive elements (GREs), TDO2 can be upregulated by glucocorticoids [21]. However, plasma glucocorticoid levels were reported to be practically the same in hepatocarcinoma patients and healthy controls or portal hypertension patients without hepatocarcinoma [22]. It is also possible that the abnormal expression of 11*β*-HSD1 and 11*β*-HSD2 in HCC, two critical enzymes that regulate the activity of glucocorticoids in the liver, accounts for the loss of TDO2 expression, but this hypothesis needs further investigation [23, 24].

## 5. Conclusions

In summary, our data demonstrate that TDO2 expression is reduced in HCC, and low TDO2 expression was correlated with a poor prognosis and adverse clinical outcomes. At the same time, TDO2 overexpression could restrain the proliferation of HCC cells in vivo and in xenograft mouse models in vitro. TDO2 overexpression induced cell cycle arrest by upregulating the expression of p21 and p27. These data indicate that TDO2 might serve as a biomarker for HCC.

## Figures and Tables

**Table 1:** The relationship between TDO2 expression and clinicopathological features in 116 patients with primary HCC.

Features	Total	TDO2 expression		*P* value
		Normal expression	Low expression	
Sex				
Male	95	43	52	0.324
Female	21	12	9	
Age (years)				
≤50	52	23	31	0.254
>50	64	34	30	
ALT				
<70	106	49	57	0.615
≥70	10	6	4	
AST				
<70	101	47	54	0.830
≥70	15	8	7	
Serum AFP (ng/ml)				
<400	62	25	37	0.101
≥400	54	30	24	
Tumor size (cm)^∗^				
≤5	39	26	13	0.011
>5	77	32	45	
Tumor number				
Single	90	59	41	0.127
Multiple	26	11	15	
BCLC stage				
0+A	75	48	27	0.019
B+C	41	17	24	
TNM stage				
I + II	86	46	40	0.027
III + IV	30	9	21	
Differentiation				
Well/moderate	73	40	33	0.038
Poor	43	15	28	
Vascular invasion				
Yes	23	7	16	0.069
No	93	48	45	
Tumor capsule				
Absent	55	26	29	0.977
Present	61	29	32	
Recurrence				
Yes	58	22	36	0.041
No	58	33	25	

## Data Availability

The data that support the findings of this study are available from the corresponding author upon reasonable request.
